# Supplementary material for: Identification of photoperiod-induced specific miRNAs in the adrenal glands of Sunite sheep (Ovis aries)
Source: Front Vet Sci. 2022 Jul 22;9:888207. doi: 10.3389/fvets.2022.888207 (PMC9354845; doi:10.3389/fvets.2022.888207)
Supplement: Supplementary file 1 [file Data_Sheet_1.ZIP › Supplementary file/miRDeep2 file.zip!/miRDeep2 file/Know_Structure/SPLP42A2/oar-mir-218a.pdf]

miRBase precursor : oar-mir-218a
Total read count : 46221
oar-miR-218a read count : 46221
remaining reads : 0

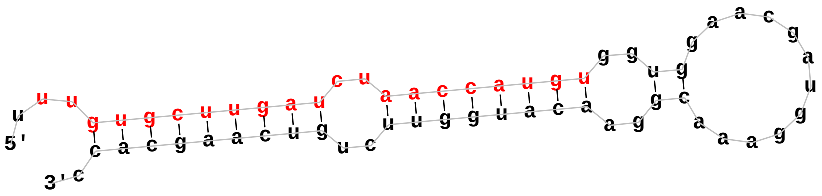

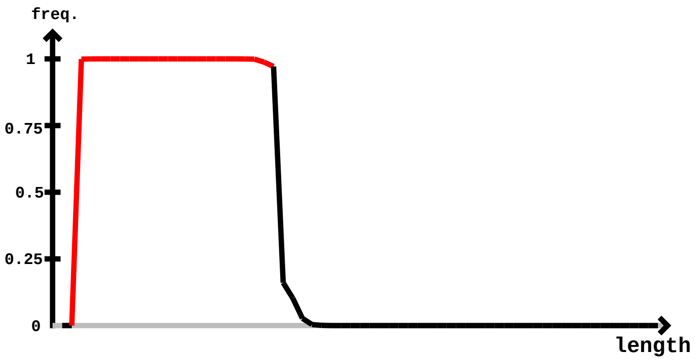

oar-miR-218a

5'- uuugugcuugaucuaaccauguggguggaacgauggaaacggaacaugguucugucaagcacc -3' exp

...((((((((..((((((((..((............))..))))))))..))))))))).	reads	mm	sample
uuugugcuugaucuaaccaugu..	6	0	seq
.uugugcuugaucuaacA..	2	1	seq
.uugugcuugaucuaacc..	14	0	seq
.uugugcuugaucuaacca...	29	0	seq
.uugugcuugaucuaaccau..	515	0	seq
.uugugcuugaucuaacAau..	1	1	seq
.uugugcuugaucuaacAaug...	2	1	seq
.uugugcuugaucuaaccaug...	757	0	seq
.uugugcuugaucuaacAaugu..	90	1	seq
.uugugcuugaucuaaccaugu..	37365	0	seq
.uugugcuugaucuaaccaugug.......................................	2686	0	seq
.uugugcuugaucuaaccaugugg......................................	3465	0	seq
.uugugcuugaucuaaccauguggu.....................................	1068	0	seq
.uugugcuugaucuaaccauguggug....................................	15	0	seq
.uugugcuugaucuaaccaugugguU....................................	127	1	seq
.uugugcuugaucuaaccaugugguUg...................................	39	1	seq
..ugugcuugaucuaaccaugu..	6	0	seq
..ugugcuugaucuaaccaugugg......................................	26	0	seq
..ugugcuugaucuaaccauguggu.....................................	4	0	seq
...gugcuugaucuaaccaugu..	4	0	seq
